# *Brachyspira suanatina* sp. nov., an enteropathogenic intestinal spirochaete isolated from pigs and mallards: genomic and phenotypic characteristics

**DOI:** 10.1186/s12866-015-0537-y

**Published:** 2015-10-12

**Authors:** Mamoona Mushtaq, Saima Zubair, Therese Råsbäck, Erik Bongcam-Rudloff, Désirée S. Jansson

**Affiliations:** Department of Animal Breeding and Genetics, Global Bioinformatics Centre, Swedish University of Agricultural Sciences (SLU), SE750 07 Uppsala, Sweden; Department of Bacteriology, National Veterinary Institute (SVA), SE751 89, Uppsala, Sweden; Department of Animal Health and Antimicrobial Strategies, National Veterinary Institute (SVA), SE751 89, Uppsala, Sweden; Department of Biomedical Sciences and Veterinary Public Health, Swedish University of Agricultural Sciences, SE750 07, Uppsala, Sweden

**Keywords:** *Brachyspira suanatina* sp. nov., Core genome, DNA-DNA hybridization, Enteropathogen, Housekeeping genes phylogeny, Spirochaete, Mallard, Phenotype, Pig, Whole genome sequence

## Abstract

**Background:**

The genus *Brachyspira* currently encompasses seven valid species that colonize the intestines of mammals and birds. In a previous study a group of strongly haemolytic isolates from pigs and mallards was provisionally described as a new species within genus *Brachyspira*, “*B. suanatina*”, and enteropathogenic properties were demonstrated in a porcine challenge model.

**Methods:**

In the current study characterization of *B. suanatina* was performed on the basis of cell morphology, growth characteristics, enzyme profiles, DNA-DNA hybridization (DDH) and whole genome comparisons. The draft genome sequence of *B. suanatina* strain AN4859/03 was determined and compared with the available genomes of all valid species of *Brachyspira*.

**Results:**

According to morphological traits, growth characteristics and enzymatic profiles, *B. suanatina* was similar to the type strain of *B. hyodysenteriae*, but using the recommended threshold value of 70 % similarity by DDH it did not belong to any of the recognized *Brachyspira* species (range 16–64 % similarity). This was further supported by average nucleotide identity values. Phylogenetic analysis performed using housekeeping genes and core genomes of all valid *Brachyspira* sp. and “*B. hampsonii*” revealed that *B. suanatina* and *B. intermedia* formed a clade distinct from *B. hyodysenteriae*. By comparing the genomes of the three closely related species *B. intermedia*, *B. hyodysenteriae* and *B. suanatina* similar profiles of general genomic features and distribution of genes in different functional categories were obtained. However, the genome size of *B. hyodysenteriae* was smallest among the species, suggesting the possibility of reductive evolution in the divergence of this species. A bacteriophage region and a putative plasmid sequence were also found in the genome of *B. suanatina* strain AN4859/03.

**Conclusions:**

The results of our study suggest that despite being similar to *B. hyodysenteriae* phenotypically, *B. suanatina* should be regarded as a separate species based on its genetic characteristics. Based on characteristics presented in this report we propose that strains AN4859/03, AN1681:1/04, AN2384/04 and Dk12570-2 from pigs in Sweden and Denmark, and strains AN3949:2/02 and AN1418:2/01 isolated from mallards in Sweden, represent a unique species within genus *Brachyspira*. For this new species we propose the name *B. suanatina* for which the type strain is AN4859/03^T^ (=ATCC® BAA-2592™ = DSM 100974^T^).

**Electronic supplementary material:**

The online version of this article (doi:10.1186/s12866-015-0537-y) contains supplementary material, which is available to authorized users.

## Background

The advent of next generation sequencing technologies with their low cost, high throughput and rapid speed has strongly influenced modern taxonomy. Many taxonomists therefore turn towards integrating genome sequence analysis for formal proposals of new bacterial taxa or reclassification of old taxa [1–3]. Early classification methods were based solely on phenotypic characteristics, but the introduction of DNA-DNA hybridization (DDH) [4, 5] and 16S ribosomal RNA (rRNA) gene phylogeny [6] in taxonomy changed the species definition [7–9]. However, due to the high level of conservation of the 16S rRNA gene [10] and the labour intensive and error prone nature of DDH experiments, there has been a constant demand for advanced methods at complete genome level. Average nucleotide identity (ANI) [11–13], multi locus sequence analysis of conserved genes [8, 14] and phylogenetic analysis of core genomes [15] are some of the suggested methods.

*Brachyspira* species are gGram-negative, anaerobic but oxygen-tolerant spirochaetes that colonize the intestines of many mammals and birds [16]. It forms a clade within phylum *Spirochaetes* that has undergone repeated taxonomic changes over the past few decades. Initially the isolates colonizing pigs were allocated to genus *Treponema* [17, 18], however they were later transferred to genus *Serpula* and then *Serpulina* [19, 20]. In 1997, the genera *Serpulina* and *Brachyspira* were unified leading to the reclassification of *Serpulina* species [21]. Genus *Brachyspira* now encompasses seven valid species: *B. aalborgi*, *B. alvinipulli*, *B. hyodysenteriae*, *B. intermedia*, *B. innocens*, *B. murdochii* and *B. pilosicoli* with *B. aalborgi* as a type species [22]. These species vary in terms of their host range and enteropathogenicity. *Brachyspira hyodysenteriae* and *B. pilosicoli* are well known porcine pathogens [16]. Furthermore, *B. pilosicoli* can cause gastrointestinal disease and egg-production losses in chickens [23], and it can also colonize the human intestine leading to human intestinal spirochaetosis [24]. However, most cases in humans are associated with *B. aalborgi,* at least in industrialized countries [25]. Moreover, *B. alvinipulli* and *B. intermedia* cause diarrhoea in chickens [23, 26]. However, *B. intermedia*, is in general considered as a harmless commensal in pigs similar to *B. innocens* and *B. murdochii* [16]. There are several proposed species without current standing in nomenclature, including “*B. suanatina*” [27].

Some members of the genus *Brachyspira* have low interspecies 16S rRNA gene variability that does not always support species delineations, especially among the indole test-positive species *B. hyodysenteriae*, *B*, *intermedia* and *B. suanatina* that require additional methods for species confirmation [27, 28]. The proposed new species *B. suanatina* was first isolated from pigs with dysentery-like disease in Sweden and Denmark, and the enteropathogenicity of strain AN4859/03 was confirmed by experimental challenge in pigs [27]. The phenotypic characterization based on the intensity of β-haemolysis and enzyme activities showed that these isolates were indistinguishable from *B. hyodysenteriae*, but detection by a species-specific PCR was unsuccessful [27]. Based on 16S rRNA and NADH oxidase (*nox*) gene similarities, these *Brachyspira* isolates from pigs were similar to some isolates from mallards [29] and were distinct from all currently recognized *Brachyspira* species, suggesting that they were members of a putatively unrecognized species. Available data suggest that *B. suanatina* most likely is a rare genotype in both pigs and mallards, and it has so far only been isolated in northern Europe. Identification of *B. suanatina* currently relies on *nox* gene sequencing and/or nox-RFLP [30]. The objective of the present study was to identify the taxonomical position of *B. suanatina* based on phenotypic characteristics and complete genome analyses*.* We have used a “taxono-genomics” approach that is based on the polyphasic combination of phenotypic and genomic characteristics [31].

## Methods

### Bacterial cultures and growth conditions

Type and reference strains *B. hyodysenteriae* B78^T^ (ATCC 27164), B204^R^ (ATCC 31212), *B. intermedia* PWS/A^T^ (ATCC 51140), *B. pilosicoli* P43/6/78^T^ (ATCC 51139), *B. innocens* B256^T^ (ATCC 29796), *B. murdochii* 56-150^T^ (ATCC 51284), *B. alvinipulli* C1^T^ (ATCC 51933) and *B. aalborgi* 513A^T^ (ATCC 43994) were originally obtained from the American Type Culture Collection (ATCC, Manassas, VA, USA) and stored in liquid nitrogen in the *Brachyspira* strain collection of the National Veterinary Institute (SVA). *Brachyspira suanatina* strains AN4859/03, AN1681:1/04, AN2384/04 and Dk12570-2 from pigs, and AN3949:2/02 and AN1418:2/01 from mallards were isolated in Sweden and Denmark as previously described [27] and stored in liquid nitrogen. The bacteria were retrieved from storage and were grown anaerobically (atmosphere generated by Anaerogen sachets, Oxoid) on fastidious anaerobe agar (FAA) (LabM) plates with 10 % equine blood at 42 °C for 72 h and subcultured once for 48 h before use. Cells for electron microscopy were harvested from broth cultures grown anaerobically on a shaker at 37 °C to mid log phase in brain heart infusion culture broth (BHI) (Difco) with 10 % foetal calf serum (FCS) (Biochrome AG) (BHIS). FAA plate cultures and broth culture were used to test the capability of all *B. suanatina* strains to grow at 32, 37, 42 and 46 °C. For the generation of growth curves, broth cultures of *B. suanatina* strains AN4859/03 and AN3949:2/02 and *B. hyodysenteriae* B78^T^ were prepared in triplicates in 10 mL BHIS at 10^6^ cells/mL and grown at 37 °C. Population densities were determined every 4 h between 11 and 70 h of incubation by colony forming unit (CFU) counts obtained from serial dilutions on FAA plates in duplicates and by spectrophotometry (OD_620_). Comparisons were made against an in-house standard curve for *B. hyodysenteriae* B78^T^. Purity was confirmed throughout the studies by phase contrast microscopy.

### Biochemical phenotype and substrate utilization

The API-ZYM test (bioMérieux) was applied to all *B. suanatina* strains with *B. hyodysenteriae* B78^T^ and *Pseudomonas aeruginosa* (ATCC 27853) as control strains. Bacterial cells were harvested from FAA plates incubated at 42 °C for 3 days, suspended in 2 mL sterile ddH_2_O to a density of 5–6 McFarland and the API-ZYM test was performed as described by the manufacturer. The test strips were incubated for 4 h in aerobic atmosphere.

*Brachyspira suanatina* strains AN4859/03, AN3949:2/02 and *B. hyodysenteriae* strain B78^T^ were used for growth substrate utilization studies. All six *B. suanatina* strains were tested for growth in D-mannitol because of diverging results obtained with strains AN4859/03 and AN3949:2/02. Potential substrates were added at 0.4 wt/v in heart infusion culture broth (HI) (Bacto™) and autoclaved or sterile filtered. The FCS concentration in the HI was selected at 7 % (v/v) from test runs at 5, 7 and 10 % FCS as described previously [26, 32]. Triplicate flasks with 10 mL HI and 7 % FCS culture broth (HIS) were used for each isolate and substrate, and were inoculated with 10^6^ cells from a stock solution made from 48 h old FAA cultures. Cultures were incubated anaerobically at 37 °C on a shaking platform. Glucose was used as positive control and HIS without added substrate as negative control. Growth was determined spectrophotometrically (OD_620_) at 3, 6 and 9 days post inoculation for all cultures, and the purity, cell density and the condition of the cells were examined by phase contrast microscopy. Compounds producing ≤0.3 OD_620_ were not considered to be growth substrates. These cultures contained degenerated and/or non-motile cells as determined by phase contrast microscopy. Cultures producing ≥0.4 OD_620_ values and moderate to profuse growth of motile spirochaetes on at least one occasion were considered to be growth substrates. The following compounds were investigated: D-glucose, D-fructose, D-fucose, D-ribose, D-mannose, sucrose, lactate, D-xylose, D-trehalose, D-galactose, D-melobiose, D-cellobiose, L-arabinose, L-fucose, L-rhamnose, D-raffinose, D-galactosamine, N-acetyl-D-glucosamine, N-acetyl-D-galactosamine, mannitol, pyruvate, esculin, porcine gastric mucin, 50x MEM amino acids, pectin, hyaluronic acid, glutathione, poly-galacturonic acid, cellulose, glycerol, succinate and glycogen.

### Electron microscopy

Strain AN4859/03 was grown in BHIS to mid-log phase, centrifuged at 14,000 × g for 3 min and washed twice in 1 mL 0.01 M sodium phosphate buffer at pH 7.0. The pellet was resuspended in 0.1 mL each of sterile ddH_2_O and negatively stained with 0.1 mL 2 % phosphotungistic acid at pH 7.0. The cells were dropped on grids and examined. For scanning electron microscopy the cells were harvested and washed as described above, dropped on grids and coated with gold-palladium with standard procedures. The samples were examined with a JSM 820 scanning electron microscope (JEOL Skandinaviska AB, Sweden). Ten cells or more were examined to determine cell dimensions, the location of attachment sites of periplasmatic flagella and the number of flagella per cell.

### DNA-DNA hybridization

The DDH of *B. suanatina* strain AN4859/03 was performed with all the seven recognized *Brachyspira* species. Cells were grown to mid-log phase in BHIS, checked for purity and centrifuged at 4 °C at 14,000 g followed by washing in PBS twice before mixing with 1:1 v/v iso-propanol (Sigma). DNA isolation and DDH were performed by DSMZ GmbH Deutsche Sammlung von Mikroorganismen und Zellkulturen GmbH DNA (DSMZ GmbH, Braunschweig, Germany). DNA was extracted using a French pressure cell (Thermo Spectronic) and was purified by chromatography on hydroxyapatite as described [33]. DNA-DNA hybridization was performed as previously described [34] using a model Cary 100 Bio UV/VIS-spectrophotometer equipped with a Peltier-thermostatted 6x6 multicell changer and a temperature controller with *in situ* temperature probe (Varian).

### DNA isolation and complete genome sequencing

Genomic DNA for sequencing of strain AN4859/03 was obtained from 60 mL mid-log BHIS cultures using the DNeasy Blood and Tissue Kit (Qiagen) as described by the manufacturer. The genome sequence was obtained using a combination of Ion torrent and Illumina MiSeq sequencing techniques performed at Uppsala Genome Centre (http://www.igp.uu.se/facilities/genome_center/). Single end reads were generated with the read size 300 bp on the Ion Torrent platform and paired end reads were generated using the Illumina MisSeq platform producing reads of 300 bp. Genomic sequences of available strains of currently valid *Brachyspira* species and two strains of the proposed species “*B. hampsonii*” were obtained from GenBank (https://www.ncbi.nlm.nih.gov/). Strain names and accession numbers are provided in Additional file 1: Table S1. Draft genome scaffolds for *B. aalborgi* were downloaded from the MetaHit website (http://www.sanger.ac.uk/resources/downloads/bacteria/metahit/).

### Genome assembly and average nucleotide identity

Single end reads generated by the Ion torrent platform were assembled using the Roche GS assembler. The resulting contigs were oriented using the *B. hyodysenteriae* WA1 genome as a reference and joining was performed manually. Reads obtained from Illumina MiSeq paired end sequencing were assembled using Mira version 4 [35]. Before performing the assembly, the reads were trimmed and filtered based on quality, using PRINSEQ [36] and further reduction in the coverage was performed using a custom perl script. Scaffolds were obtained using SSPACE [37]. The scaffolds were aligned to Ion torrent assembly using MAUVE [38] to find possible overlaps. Overlapping scaffolds were manually joined in Consed [39]. A putative plasmid sequence was predicted by blasting the *B. hyodysenteriae* WA1 plasmid’s (pBHWA1) sequence [40] against the *B. suanatina* contigs.

The average nucleotide identity (ANI) between *B. suanatina* and type strains of all the seven valid *Brachyspira* species was calculated using Jspecies v1.2.1 [13] using BLAST (ANIb) and MUMMER (ANIm) algorithms with default parameters.

### Genome annotation and core genome analysis

*B. suanatina* scaffolds were annotated using the GenDB platform [41]. Predictions made by the GenDB pipeline were also manually reviewed and curated. Prophages were predicted using PHAST [42] and by manual curation in the annotated genome. Clusters of orthologous group (COG) categories were assigned to genes by performing blast search against the COG database (http://www.ncbi.nlm.nih.gov/COG) using the threshold of > =30 % amino acid identity and e-value < = 0.00005. In order to obtain annotations based on the latest versions of search databases, all the genomes used in the analysis were also annotated with GenDB. Comparative analysis of the *B. suanatina* genome with other *Brachyspira* genomes including pan and core genome calculations was performed using the EDGAR software framework for prokaryotic genomes [43]. Whole genome alignment of *B. suanatina* with the complete genomes of *B. hyodysenteriae* WA1 and *B. intermedia* PWS/A^T^ was performed using the Artemis comparison tool (ACT). Phylogenetic analysis based on core genomes of all *Brachyspira* species including *B. suanatina* was also performed as a part of the EDGAR comparative analysis. For phylogenetic analysis, multiple sequence alignments of amino acid sequences of the core genome were obtained in MUSCLE [44], unaligned blocks were removed using GBLOCKS [45] and a phylogenetic tree of concatenated alignment was generated in PHYLIP using the neighbour joining method. Bootstrap analysis was performed on the tree using 200 (bootstrap) replicates.

### Phylogenetic analysis of housekeeping genes

For phylogenetic analysis of housekeeping genes, 31 protein coding genes that are universally conserved among bacteria [46] were selected (*dnaG*, *frr*, *infC*, *nusA*, *pgk*, *pyrG*, *rplA*, *rplB*, *rplC*, *rplD*, *rplE*, *rplF*, *rplK*, *rplL*, *rplM*, *rplN*, *rplP*, *rplS*, *rplT*, *rpmA*, *rpoB*, *rpsB*, *rpsC*, *rpsE*, *rpsI*, *rpsJ*, *rpsK*, *rpsM*, *rpsS*, *smpB*, and *tsf*). Nucleotide sequences of these genes for *B. hyodysenteriae* B78^T^, *B. hyodysenteriae* WA1, *B. intermedia* PWS/A^T^, *B. murdochii* 56-150^T^, *B. pilosicoli* 95/1000, *B. pilosicoli* B2904, *B. pilosicoli* WesB, *B. pilosicoli* P43/6/78^T^, *Treponema denticola* a^T^ and *Borrelia Burgdorferi* B31^T^ were obtained from the NCBI Gene database (http://www.ncbi.nlm.nih.gov/gene). Nucleotide sequences for *B. aalborgi* 513A^T^, *B. alvinipulli* C1^T^, *B. innocens* B256^T^, “*B. hampsonii*” 30446, “*B. hampsonii*” 30599 and *B. suanatina* AN4859/03 were predicted by performing homology searches against their gene sequences with *B. hyodysenteriae* strain WA1’s genes as reference. Sequences were aligned using MUSCLE [44] and conserved blocks were obtained using Gblocks [45]. Genes with < 50 % alignment conservation were removed leaving 26 genes. Phylogenetic inference using the concatenated alignment of those 26 genes was drawn using the maximum likelihood method (ML) [47] with 1000 bootstrap replicates. The general time reversible (GTR) [48] model with discrete gamma distribution allowing some proportion of invariable sites (+G + I) as predicted by JModelTest [49] was used to model the evolutionary rates among sites. Phylogenetic analysis was performed in MEGA6, an evolutionary analysis tool [50].

## Results

### Morphology and growth characteristics

Cells of strain AN4859/03 were ultrastructurally similar in appearance but slightly shorter and thinner and possessed fewer flagella than those of *B. hyodysenteriae* B78^T^ and *B. intermedia* PWS/A^T^ (Table 1). The cells were shaped as spirochaetes with tapered ends and periplasmic flagella that were inserted on a line subterminally at both cell ends.

**Table 1:** Morphologic comparison of indole-positive species of genus *Brachyspira* ^a, b^

Species	Strains used	Cell length (μm)	Cell diameter (μm)	No. of flagella/cell
*B. hyodysenteriae*	B78^T^	9.78 ± 1.87	0.35 ± 0.02	16 – 24
		8 – 10	0.3 – 0.4	14 – 18
		6 – 8.5	0.35 – 0.4	24 – 28
*B. intermedia*	PWS/A^T^	7.5 – 10	0.35 – 0.45	24 – 28
*B. suanatina*	AN4859/03	5.5 ± 1.0	0.3 ± 0.0	14 – 16

^a^Results for B*B*. *hyodysenteriae* and B*B*. *intermedia* from previous studies [21, 32, 61]

^b^Results from present study

Strains AN4859/03 and AN3949:2/02 that were cultured anaerobically in BHIS broth at 37 °C entered the log-phase at approximately 31 h of incubation and reached maximum population densities at 36–38 h post inoculation. Maximum CFU/mL was 2 × 10^8^ cells/mL at 36 h. At mid log-phase, the population doubling time was approximately 2 h. Both strains grew equally well at 37 and 42 °C, but growth was inhibited at 32 and 46 °C.

### Biochemical tests

The enzymatic profiles are shown in Table 2. All six *B. suanatina* isolates had a similar enzymatic profile to *B. hyodysenteriae strain* B78^T^, but reactions were generally weaker.

**Table 2:** Enzymatic profile of *B. hyodysenteriae* type strain (B78^T^) and six strains of *B. suanatina*

Strain	*Brachyspira panel* ^*a*^					api-ZYM enzyme activities^b^																			
	Haem	IS	Hipp	α-gal	β-glu	1	2	3	4	5	6	7	8	9	10	11	12	13	14	15	16	17	18	19	20
B78^T^	s	+	–	–	+	0	4	3	1	0	0	0	0	1	2	4	1	1	5	2	4	4	0	0	0
AN4859/03	s	+	–	–	+	0	2	1	1	0	0	0	0	0	0	2	1	0	5	0	2	4	0	0	0
AN1681:1/04	s	+	–	–	+	0	2	1	1	0	0	0	0	0	0	2	1	0	4	0	2	3	0	0	0
AN2384/04	s	+	–	–	+	0	2	1	1	0	0	0	0	0	0	1	2	0	5	0	2	4	0	0	0
Dk12570-2	s	+	–	–	+	0	3	1	1	0	0	0	0	0	0	2	1	0	5	0	2	3	0	0	0
AN3949:2/02	s	+	–	–	+	0	3	1	1	0	0	0	0	0	0	2	1	0	5	0	2	3	0	0	0
AN1418:2/01	s	+	–	–	+	0	2	1	1	0	0	0	0	0	0	3	1	0	5	0	3	4	0	0	0

^a^ *Brachyspira* panel according to Fellström et al., 1999. Results according to Råsbäck et al., 2007. Haem = intensity of β-haemolysis; IS = indole spot test; Hipp = hippurate cleavage capacity; α-galactosidase; β-glucosidase; s = strong reaction; + = positive reaction, − = negative reaction

^b^API-ZYM test: 1 control; 2 alkaline phosphatase; 3 C_4_ esterase; 4 C_8_ esterase lipase; 5 C_14_ lipase; 6 leucine arylamidase; 7 valine arylamidase; 8 cystine arylamidase; 9 trypsin; 10 chymotrypsin; 11 acid phosphatase; 12 naphtolphosphohydrolase; 13 α-galactosidase; 14 β-galactosidase; 15 β-glucoronidase; 16 α-glucosidase; 17 β-glucosidase; 18 N-acetyl-β-glucosaminidase; 19 α-mannosidase; 20 α-fucosidase; values 0–5 assigned according to the manufacturer’s instruction 0 = negative reaction, 5 = maximum intensity

### Growth substrate utilization

The maximum growth (median OD_620_ values of three replicates) of strains *B. hyodysenteriae* B78^T^ and *B. suanatina* strains AN4859/03 and AN3949:2/02 in HIS but without added growth substrate was 0.2–0.3. The following compounds were utilized by all three strains as growth substrates: D-glucose, D-fructose, D-galactose, D-mannose, sucrose, D-trehalose, lactose, N-acetyl-D-glucosamine, pyruvate (OD_620_ ≥ 0.4–1.0). Three out of six tested *B. suanatina*-strains (AN3949:2/02, AN14 18:2/01 and Dk12570) metabolized D-mannitol, but *B. hyodysenteriae* B78^T^ did not. Substrates that were not metabolized by the three tested strains included: D-cellobiose, D-fucose, D-melobiose, D-ribose, D-raffinose, D-xylose, L-arabinose, L-fucose, L-rhamnose, N-acetyl-D-galactosamine, soluble starch, galactosamine, Na-acetate, lactate, esculin, succinate, pectin, 50x MEM amino acids, cellulose, glutathione, glycerol, glycogen, poly-galacturonic acid, hyaluronic acid and hog gastric mucin. The control strain B78^T^ showed results consistent with an earlier study [32].

### DNA-DNA hybridization

The percent DNA-DNA similarities of *B. suanatina* strain AN4859/03 to other *Brachyspira* species type strains are given in Table 3.

**Table 3:** Percent DNA-DNA similarity of *B. suanatina* AN4859/03 to type strains (duplicated results) and percent ANIb/ANIm values

*Brachyspira sp.*	Strain designation	ATCC no.	% DNA-DNA similarity^a^	ANIb/ANIm values
*B. hyodysenteriae*	B78^T^	27164	62.9/64.0	92.12/92.61
*B. intermedia*	PWS/A^T^	51140	59.0/64.0	92.69/93.19
*B. pilosicoli*	P43/6/78^T^	51139	22.1/27.9	79.29/86.36
*B. innocens*	B256^T^	29796	19.9/16.2	82.78/86.08
*B. murdochii*	56-150^T^	51284	35.7/31.0	82.78/85.98
*B. alvinipulli*	C1^T^	51933	49.9/58.9	83.81/87.01
*B. aalborgi*	513A^T^	43994	34.1/31.0	75.28/82.17

^a^in 2 X SSC at 59 °C

### Assembly statistics and Average nucleotide identity

The final assembly of *B. suanatina* AN4859/03 consisted of 34 scaffolds comprising 3,258,009 bp with a GC content of 27 %. Three large scaffolds of sizes 2,243,936 bp, 499,175 bp and 433,837 bp covered 98 % of the assembled genome. The remaining scaffolds ranged in size from 450 bp to 30,000 bp. One of the scaffolds contained a putative plasmid sequence of 30,236 bp sharing 88 % identity over 51 % of its length with the *B. hyodysenteriae* strain WA1 plasmid (pBHWA1) sequence, see below. The assembled genome of *B. suanatina* strain AN4859/03 was larger than that of the available genomes of *B. hyodysenteriae* strains B78^T^ and WA1, but smaller than the genome of *B. intermedia* strain PWS/A^T^. The ANI of *B. suanatina* to type strains of other *Brachyspira* species was lower than 95 %, with the highest value obtained between *B. suanatina* and *B. intermedia* (Table 3).

### General genomic features and putative plasmid

General genomic features of *B. suanatina* strain AN4859/03, *B. hyodysenteriae* (strains B78^T^ and WA1) and *B. intermedia* (PWS/A^T^) are depicted in Table 4. The draft genome assembly of *B. suanatina* AN4859/03 consisted of 2,892 open reading frames (ORFs), including 30 ORFs (BRSU_2794–BRSU_2823) belonging to a putative plasmid sequence. By comparing the plasmid genes of *B. suanatina* with that of pBHWA1, we found 19 CDSs that were conserved in both strains. These include four genes encoding for epimerases, six genes encoding for transferases, and one gene each encoding for radical sterile alpha motif (SAM) domain containing protein, plasmid partition protein, putative replicative DNA helicase, DNA primase-like protein, integrase, lipopolysaccharide biosynthesis protein, dehydratase, reductase and a hypothetical protein. Genes that were present in *B. suanatina* but were not found in the plasmid sequence of *B. hyodysenteriae* were one glycosyl transferase, one peptide ABC transporter substrate-binding protein, one radical SAM domain-containing protein, five hypothetical proteins, one conserved hypothetical protein and two of unknown function.

**Table 4:** Genome assembly statistics^a, b^: Summary of general genomic features of *B. suanatina* strain AN4859/03 and its comparison with genomes of *B. hyodysenteriae* strains B78^T^ and WA1 and *B. intermedia* strain PWS/A^T^

Genomic feature	*B. suanatina AN4859/03*	*B. hyodysenteriae B78* ^*T*^	*B. hyodysenteriae WA1*	*B. intermedia PWS/A* ^*T*^
Total Assembly size (bp)	3,258,009	3,046,380	3,036,634	3,308,048
Number of contigs	35	46	2	2
Chromosome				
Size (bp)	3,227,373	3,046,380	3,000,694	3,304,788
DNA GC %	27	27	27.06	27.22
Protein coding genes	2,826	2,593	2,613	2,870
Plasmid				
Size (bp)	30.236	-	35,940	3,260
DNA GC %	22.14	-	21.82	21.00
Protein coding genes	30	-	31	3
Number of predicted tRNA genes	33	33	33	33
Number of predicted rRNA genes	3	3	3	3
Number of prophages	2	0	0	2
Genes assigned to COG categories	2,207	2,105	2,104	2,224

^a^Data from references [40, 53] and from the present study (B*B*. *suanatina*, strain AN4859/03)

^b^B*B*. *hyodysenteriae* B78^T^ data taken from ncbi WGS assembly GCF_000383255 with accession NZ_ARSY00000000

### Prophages and mobile genetic elements

The *B. suanatina* genome contained a 15 kb region similar to the core region of the prophage-like gene transfer agent (GTA) named VSH-1 (Virus of *Serpulina hyodysenteriae*) of *B. hyodysenteriae* (BRSU_2672 – BRSU_2703). By comparing this region with the GTA region of *B. hyodysenteriae* strain WA1 (GTA/Bh-WA1) [51], we found that all the late function genes were present in the same order in both strains. Of the twelve ORFs of unknown function described in *B. hyodysenteriae* WA1’s GTA region, *orfA*, *orfB*, *orfE*, *orfF*, *orfG*, *orf1*, *orf5* and *orf6* were conserved in *B. suanatina*, and *orfC*, *orf2*, *orf3* and *orf4* were not present in the *B. suanatina* genome. Instead six other ORFs of unknown function were found in the corresponding region in *B. suanatina*. Two of them were also conserved in the VSH-1 regions of *B. hyodysenteriae* B78^T^ (ARSY01_298 and ARSY01_299) and *B. intermedia* PWS/A^T^ (Bint_0120 and Bint_0123). One of them was homologous to a gene encoding for sulfatase (Bint_1870) outside the VSH-1 region in *B. intermedia* PWS/A^T^ and three of them were unique to the *B. suanatina* genome. Besides these six ORFs, three additional ORFs were present between *hvp45* and *hvp19*. Two of them were also present in the VSH-1 region of *B. intermedia* encoding for hypothetical proteins and one of them was unique to *B. suanatina*. Another additional ORF was present between *hvp101* and *lys* gene in the *B. suanatina* genome and was homologous to *hvp28* gene in VSH-1 region of *B. intermedia* PWS/A^T^.

**Fig. 1:**
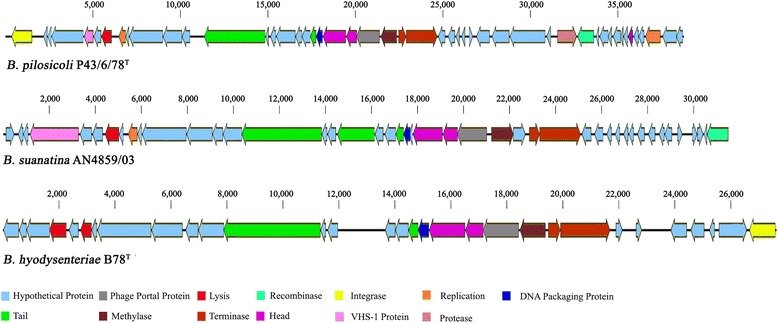
Bacteriophage region comparison: Comparison of the organization of the bacteriophage region BSP in *B. suanatina* AN4859/03 with the homologous bacteriophage regions in *B. pilosicoli* P43/6/78^T^ and *B. hyodysenteriae* B78^T^ genomes

A putative bacteriophage region, BSP was predicted in the *B. suanatina* genome, which has not been described in genomes of *B. hyodysenteriae* strain WA1 or *B. intermedia* strain PWS/A^T^. Analysis revealed that BSP was present approximately 33 kb upstream to the VSH-1 region. The BSP region spanned over a 31.2 kb sequence length and consisted of 31 CDSs (BRSU_2592–BRSU_2638) including one VSH-1 related gene. The BSP region was not present in the complete genome of *B. hyodysenteriae* strain WA1 genome, but a homologous bacteriophage region was found in scaffold number 2 of the *B. hyodysenteriae* B78^T^ assembly (NZ_ARSY00000000) and consisted of 29 CDSs (Fig. 1). A bacteriophage region almost identical to BSP was found in the *B. pilosicoli* P43/6/78^T^ genome (CP002873) spanning over 39 kb sequence and it contained 50 CDSs (BPP43_03795 to BPP43_04045) including one VSH-1 related gene. The organization of genes in BSP and its comparison with identical regions in *B. hyodysenteriae* B78^T^ and *B. pilosicoli* P43/6/78^T^ is shown in Fig. 1. Further analysis revealed that a significant portion of the BSP region was conserved and had been previously reported as pP1, pP2 and pP3, in *B. pilosicoli* 95/1000, *B. pilosicoli* strains B2904 and *B. pilosicoli* WesB respectively [52], and pM1, pM2 and pM3 in *B. murdochii* strain 56-150^T^ [53]. Some of the BSP genes were also found in genomes of *B. innocens* B256^T^ and *B. alvinipulli* C1^T^ scattered in the genome either as single genes or in small clusters of phage related genes. These include ARQI01_47, ARQI01_48, ARQI01_86, ARQI01_102, ARQI01_109, ARQI01_110, ARQI01_347-ARQI01_356, ARQI01_462-ARQI01_466, ARQI01_469-ARQI01_472, ARQI01_477, ARQI01_478 in the *B. innocens* genome (accession: NZ_ARQI00000000) and JADF01_1734, JADF01_1737, JADF01_1738, JADF01_1749, JADF01_2178, JADF01_2181, JADF01_2185, JADF01_2187, JADF01_2568, JADF01_2575, JADF01_ 2578, JADF01_2975, JADF01_2976, JADF01_2980 – JADF01_2989, JADF01_2992, JADF01_3246, JADF01_3247, JADF01_3249, JADF01_3252, JADF01_3255, JADF01_3257, JADF01_3258 in the *B. alvinipulli* genome (accession: NZ_JADF00000000). Only two genes of BSP shared homology with *B. intermedia* strain PWS/A^T^ genes. These were: Bint_1528 encoding hypothetical protein of *B. intermedia* phage and Bint_1547 encoding putative integrase in *B. intermedia*. A part from these bacteriophage regions, some phage related genes and mobile genetic elements were also found in the *B. suanatina* genome including putative integrases, terminases, tail protein, portal protein that were not found in *B. hyodysenteriae* genome except for the two reported phage related genes BHWA_01969 and BHWA_02688. Bacteriophage regions of *B. intermedia* PWS/A^T^ [53] were not found in the *B. suanatina* genome except one gene (Bint_0072) that encodes for a hypothetical membrane spanning protein, and is also conserved in *B. hyodysenteriae* strains WA1 and B78^T^.

### Gene content and synteny conservation

**Fig. 2:**
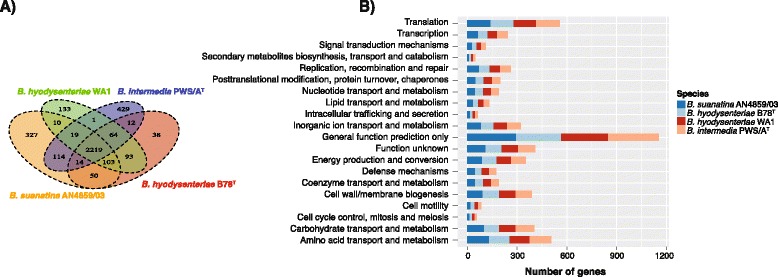
Global gene conservation and comparison of functional classification of genes: The figure shows the number of genes and their classification in different COG categories from *B. suanatina* AN4859/03, *B. hyodysenteriae* B78^T^, *B. hyodysenteriae* WA1 and *B. intermedia* PWS/A^T^ (**a**) Venn diagram (**b**) The bar chart shows the distribution of genes in different functional categories. Genes were assigned to different categories using blastP against the COG database

The pan genome of *B. suanatina* AN4859/03, *B. hyodysenteriae* B78^T^, *B. hyodysenteriae* WA1 and *B. intermedia* PWS/A^T^ consisted of 3,682 genes. Among them 2,219 genes were present in all four strains, 2,351 genes were shared between *B. hyodysenteriae* WA1 and *B. suanatina*, 2,386 genes were shared between *B. hyodysenteriae* B78^T^ and *B. suanatina,* and 2,366 genes were shared between *B. intermedia* and *B. suanatina* (Fig. 2a). The remaining genes had their highest similarities with other *Brachyspira* species. There were 83 genes in *B. suanatina* that were not present in any of the other *Brachyspira* species, most of them consisted of hypothetical proteins and some had matches in *Clostridium* spp. and *Bacillus* spp.

By comparing the functional distribution of genes in different COG categories, the four strains *B. suanatina* AN4859/03, *B. hyodysenteriae* B78^T^, *B. hyodysenteriae* WA1 and *B. intermedia* PWS/A^T^ contained similar numbers of genes for particular functions (Fig. 2b) (Additional file 2: Table S2).

In contrast to gene content, gene synteny was highly conserved between *B. hyodysenteriae* and *B. suanatina* with only two major genomic rearrangement events that were found in *B. hyodysenteriae* WA1 and *B. suanatina.* Some insertions were also found in the *B. suanatina* genome, when compared with *B. hyodysenteriae* WA1, comprising mainly of genes encoding for unknown proteins, bacteriophages and mobile genetic elements. In *B. intermedia* and *B. suanatina*, there was a low level of conservation of synteny as compared to the gene content. The alignment of genomic sequences for two isolates of *B. suanatina* and *B. intermedia* PWS/A^T^ using ACT identified extensive genomic rearrangements, including at least eight major inversions (Additional file 3: Figure S1).

### Phylogenetic inference based on the core genome and housekeeping genes

**Fig. 3:**
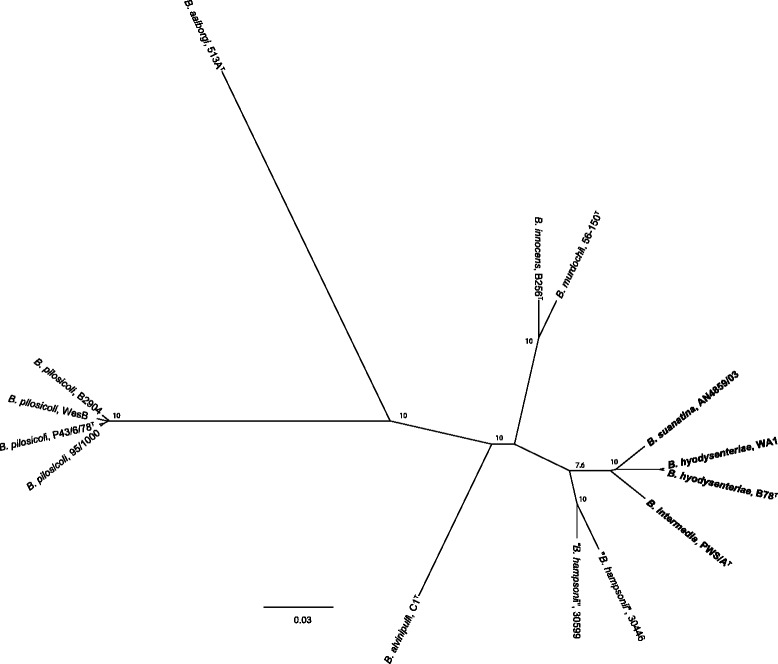
Phylogenetic tree of core genome: Radial unrooted phylogenetic representation based on concatenated amino acid sequences of 1,309 core genes of available genomes of genus *Brachyspira* and *B. suanatina* strain AN4859/03. The clade containing *B. hyodysenteriae*, *B. intermedia* and *B. suanatina* strains are shown in bold. The tree was constructed using the neighbour joining method. Numbers above the branches represent support values (>0.5) obtained from 200 bootstrap replicates. The tree is drawn to scale with branch lengths measured in the number of substitutions per site

**Fig. 4:**
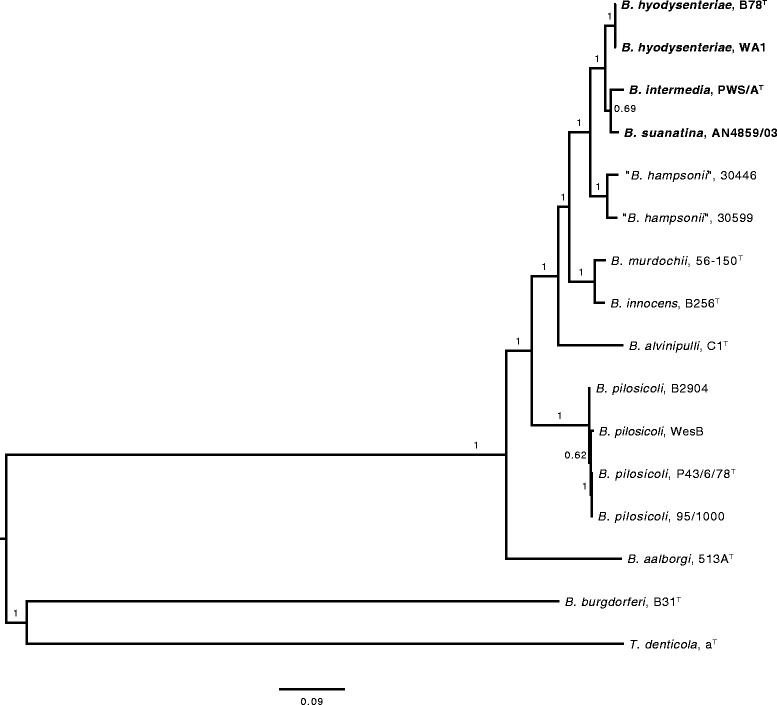
Phylogenetic tree of housekeeping genes: The evolutionary history of 26 housekeeping genes of a selection of strains representing phylum *Spirochaetes* was inferred by the maximum likelihood method [47] based on the general time reversible model [48]. A discrete Gamma distribution was used to model evolutionary rate differences among sites (+G, parameter). The model allowed some sites to be in variable (+I, parameter). The clade containing *B. hyodysenteriae*, *B. intermedia* and *B. suanatina* strains are shown in bold. *Treponema denticola* a^T^ and *Borrelia burgdorferi* B31^T^ were used as outgroup. Numbers above branches represent support values obtained from 1000 bootstrap replicates. The tree is drawn to scale with branch lengths measured in the number of substitutions per site. Evolutionary analysis was conducted in MEGA6 [50]

In order to infer the evolutionary relationship of *B. suanatina* with other *Brachyspira* species, phylogenetic analysis was performed on the amino acid sequences of core genomes and nucleotide sequences of housekeeping genes. In total 1,309 genes were found conserved in all *Brachyspira* species and their phylogenetic tree exhibited a close relationship between *B. suanatina* and *B. intermedia* whereas *B. hyodysenteriae* WA1 and *B. hyodysenteriae* B78^T^ formed a distinct clade. The phylogenetic tree is shown in Fig. 3. Phylogenetic analysis of 26 housekeeping genes also showed a highly similar tree topology to the core genome analysis and confirmed a clear distinction between *B. hyodysenteriae* and *B. suanatina*. The confidence level for the tree topology using 1000 bootstrap replicates ranged from 60 % to 100 % (Fig. 4).

## Discussion

Using the threshold value of 70 % DNA-DNA similarity for the delineation of bacterial species, as recommended by the *ad hoc* committee [9], *B. suanatina* strain AN4859/03 does not belong to any of the currently recognized seven *Brachyspira* spp. The highest observed % DNA-DNA similarities (in the range of 59.0-64.0) were to the type strains of *B. hyodysenteriae* and *B. intermedia*. Our finding was further confirmed by the ANI values, which are considered to correspond with the % DDH values [11]. Since the degree of robustness of ANIm is higher compared to ANIb for genomes having >60 % DDH values [13] we calculated the ANI values using both algorithms and the values obtained from them are in agreement to each other. Furthermore, the evolutionary history inferred from analysis of the 16S rRNA gene [27], housekeeping genes and the core genome (present study) suggests a common ancestor of the three related strains B78^T^, AN4859/03, and PWS/A^T^, which each represent indole test-positive phenotypes of *B. hyodysenteriae*, *B. suanatina* and *B. intermedia*, respectively. High bootstrap values supported the phylogram based on housekeeping genes and the assertion that *B. suanatina* and *B. intermedia* make a separate clade that is distinct from the clade formed by *B. hyodysenteriae* strains. Interestingly, the phenotypes of *B. hyodysenteriae*, *B. suanatina* and *B. intermedia* were very similar, with the strong β-haemolysis of the two former being strongly positive vs. the weak β-haemolysis in *B. intermedia*. Notably, in this study, no consistent diagnostic phenotypic feature was found to differentiate *B. suanatina* from *B. hyodysenteriae.* The strains of *B. suanatina* in this study showed varying capabilities of using D-mannitol as growth substrate, whereas strain B78^T^ could not. Even though this phenotype could be further investigated it would not qualify as a reliable diagnostic feature.

Interestingly, among the three strains, the type strain of *B. intermedia* PWS/A^T^ seems to have undergone the highest number of changes since its divergence from the common ancestor based on the housekeeping genes phylogram. When comparing the conservation of gene synteny of *B. suanatina* with *B. hyodysenteriae* WA1 and *B. intermedia* PWS/A^T^, very low conservation was found between *B. suanatina* and *B. intermedia* as compared to *B. suanatina* and *B. hyodysenteriae* WA1. These results also point to the high level of genomic rearrangements that have taken place in the genome of *B. intermedia* after these species diverged. Previous reports based on multi locus sequence typing have suggested that *B. intermedia* is a genetically diverse species that may possibly consist of several species [54, 55]. The same applies when strongly β-haemolytic indole-test positive isolates, to which *B. hyodysenteriae* and *B. suanatina* and other isolates of unknown species affiliation, are investigated [29]. Clearly, this group of *Brachyspira* isolates needs more detailed genomic and phenotypic study to elucidate evolutionary and taxonomic relationships.

General genomic features including the distribution of genes in similar functional classes in *B. suanatina*, *B. hyodysenteriae* and *B. intermedia* support the fact that they are highly similar species. However the genome size of *B. hyodysenteriae* is smaller as compared to *B. suanatina* and *B. intermedia*. Speculatively, the reason for the smaller genome size could be that *B. hyodysenteriae* has adapted to the rich environment of porcine intestinal tissues through reductive evolution. Reductive evolution is a process of reduction in genome size of a host associated bacterium by the loss of genes rendered non-essential [56]. The presence of intact bacteriophage region BSP in *B. suanatina* and its conservation in other *Brachyspira* genomes including *B. hyodysenteriae* B78^T^ while absence in *B. hyodysenteriae* WA1 and *B. intermedia* PWS/A^T^ also suggests the loss of this prophage in two later species due to the reductive evolution. Events of horizontal gene transfer (HGT) are also evident in the genome of *B. suanatina* when examining the genes that had no homology in the genomes of *Brachyspira* species with other bacteria. These include the presence of some genes that had their highest similarities in *Clostridium* spp*.* and *Bacillus* spp. Both these species are also inhabitants of the large intestine where the possible exchange of genetic material with *B. suanatina* could have taken place. Events of reductive evolution and HGT in the genomes of *Brachyspira* species have been described earlier [40, 52, 53].

The unusual phage-like gene transfer agent VSH-1 produced by *B. hyodysenteriae* is likely involved in natural gene transfer and recombination within different strains of the species [57, 58]. It spans over a 16.3 kb region of the genome of *B. hyodysenteriae* B204^R^ and contains all the late function genes [59]. The region containing genes homologous to VSH-1 has also been identified in the genomes of different strains of *B. hyodysenteriae*, *B. intermedia*, *B. pilosicoli* and *B. murdochii* [51, 53, 60]. A region similar to VSH-1 was also found in the genome of *B. suanatina* in this study. Although it is not clear if this GTA in *B. suanatina* strain AN4859/03 is functional, the conservation of the late function genes and the presence of unknown genes in the GTA region suggest functional capabilities.

## Conclusions

This study demonstrates the importance of integrating genomic data into the classification of different bacterial species. Based on genomic characteristics we conclude that *B. suanatina* does not belong to any of the currently validated seven *Brachyspira* species. The use of genomic data from more than one strain of *B. suanatina* species of the strain will facilitate future comparative genomic studies.

### Description of *Brachyspira suanatina* sp. nov.

*Brachyspira suanatina* su.a.na.ti'na. L. masc n. suus swine/pig, L. fem. adj. anatina from/of a duck, N.L. fem. adj. suanatina of pig and duck, referring to the host animals (pigs and mallards) from which this spirochete was isolated.

Cells are oxygen-tolerant but anaerobic and Gram-negative of a typical spirochaetal ultrastructure. Cell dimensions are 5.5 ± 1.0 μm in length and 0.3 ± 0.0 μm in diameter. The number of periplasmic flagella are 14–18 (mean 16) per cell inserted subterminally at cells ends. Bacterial growth occurs at 37 and 42 °C but not at 32 and 46 °C. When grown anaerobically in BHIS at 37 °C cultures reach log-phase at approximately 31 h of inoculation and reach maximum population densities of approximately 2 × 10^8^ CFU/mL at 36–38 h post inoculation. At mid log-phase, the population doubling time is approximately 2 h. Cells grow anaerobically on fastidious anaerobe agar with swarming colonies and a strong β-haemolysis on trypticase soy agar. Gives positive reactions in tests for tryptophanase activity, alkaline phosphatase, C_4_ esterase, C_8_ esterase lipase, acid phosphatase, naphtolphosphohydrolase, β-galactosidase. α-glucosidase, β-glucosidase. The following substrates can be utilized as single carbon, nitrogen and energy sources for growth: D-glucose, D-fructose, D-galactose, D-mannose, sucrose, D-trehalose, lactose, N-acetyl-D-glucosamine and pyruvate. D-mannitol sustain growth of some isolates. Growth was not detected with D-cellobiose, D-fucose, D-melobiose, D-ribose, D-raffinose, D-xylose, L-arabinose, L-fucose, L-rhamnose, N-acetyl-D-galactosamine, soluble starch, galactosamine, Na-acetate, lactate, esculin, succinate, pectin, 50x MEM amino acids, cellulose, glutathione, glycerol, glycogen, poly-galacturonic acid, hyaluronic acid and hog gastric mucin. The draft genomic DNA G + C content of the type strain is 27 % and the genome comprises 3,258,009 bp, and 2,892 ORFs. DNA-DNA hybridization of the type strain against all type strains of recognized *Brachyspira* spp. (<64.0 %) and ANIb/ANIm values (75.28-92.69 and 85.98-93.19 respectively) support the taxonomic classification of *B. suanatina* as a sp. nov. The evolutionary history of 16S rRNA and housekeeping genes, and the core genome shows a common ancestor of *B. hyodysenteriae*, *B. intermedia* and *B. suanatina*. A bacteriophage region and a phage-like GTA similar to VSH-1 in *B. hyodysenteriae* and a putative plasmid sequence related to *B. hyodysenteriae* strain WA1 plasmid (pBHWA1) are present in the genome of *B. suanatina* strain AN4859/03. Type strain is enteropathogenic in pigs with swine dysentery-like symptoms when using the type strain, and mild diarrhoea was associated with challenge with an isolate of mallard origin [27]. The type strain, AN4859/03^T^ (=ATCC® BAA-2592™ = DSM 100974^T^) was obtained from a Swedish pig with swine dysentery-like disease.

### Accession numbers

Draft genome sequences of *B. suanatina* AN4859/03 have been deposited in the European Nucleotide Archive (ENA) database with scaffold accession numbers: CVLB01000001-CVLB01000034 under the study accession number: PRJEB9032.

## Additional files

Additional file 1: Table S1. Strain names and accession numbers of available genome used for the study. (XLSX 10 kb) Additional file 2: Table S2. Functional distribution of genes in different COG categories of strains *B. suanatina* AN4859/03, *B. hyodysenteriae* B78^T^, *B. hyodysenteriae* WA1 and *B. intermedia* PWS/A^T^. (XLSX 10 kb) Additional file 3: Figure S1. Pairwise genome alignment of *B. hyodysenteriae* WA1, *B. intermedia* PWS/A^T^ and *B. suanatina* AN4859/03. The genomes were aligned using Artemis comparison tool (ACT). Alignment was performed according to the sequence start of *B. hyodysenteriae* WA1 and visualised in ACT with a cut-off set to blast scores > 250. Red bars indicate the similar regions in the same orientation, whereas blue bars show inverted regions. (PDF 662 kb)

## Abbreviations

ACT: Artemis comparison tool
ANI: Average nucleotide identity
BHI: Brain heart infusion culture broth
BHIS: Brain heart infusion culture broth with calf serum
CDS: Coding sequence
CFU: Colony forming unit
COG: Clusters of orthologous group
DDH: DNA-DNA hybridization
FAA: Fastidious anaerobe agar
FCS: Foetal calf serum
GTA: Gene transfer agent
HI: Heart infusion culture broth
HIS: Heart infusion culture broth with foetal calf serum
HGT: Horizontal gene transfer
ML: Maximum likelihood
*Nox*: NADH oxidase gene
ORF: Open reading frame
