# Supplementary figures and images for: Brachyspira suanatina sp. nov., an enteropathogenic intestinal spirochaete isolated from pigs and mallards: genomic and phenotypic characteristics
Source: BMC Microbiol. 2015 Oct 12;15:208. doi: 10.1186/s12866-015-0537-y (PMC4603578; doi:10.1186/s12866-015-0537-y)

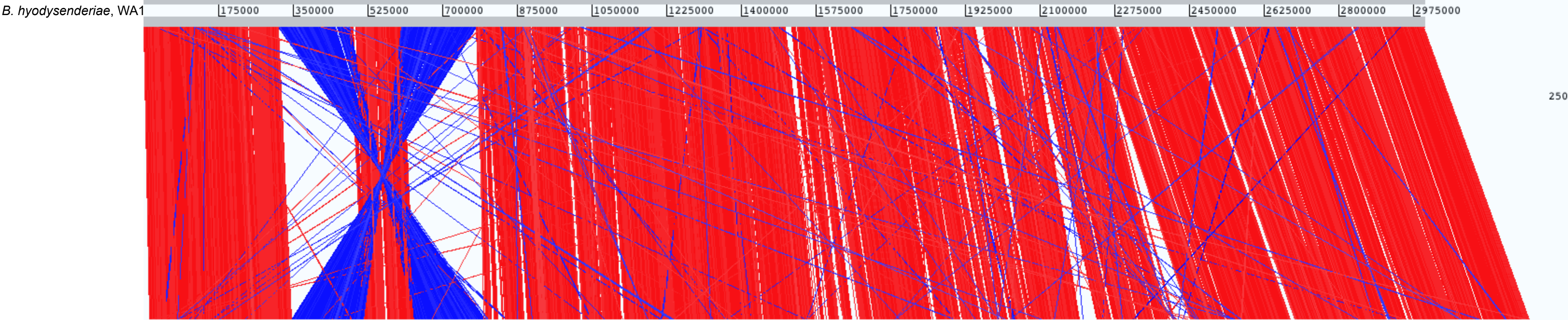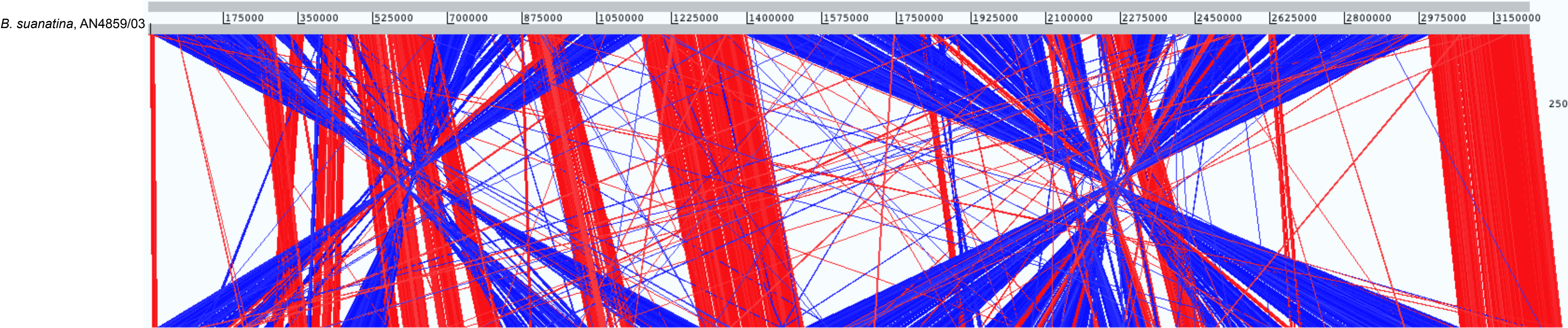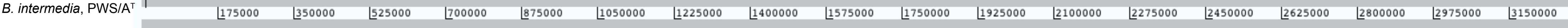

Supplement: Additional file 3: Figure S1. — Pairwise genome alignment of B. hyodysenteriae WA1, B. intermedia PWS/AT and B. suanatina AN4859/03. The genomes were aligned using Artemis comparison tool (ACT). Alignment was performed according to the sequence start of B. hyodysenteriae WA1 and visualised in ACT with a cut-off set to blast scores > 250. Red bars indicate the similar regions in the same orientation, whereas blue bars show inverted regions. (PDF 662 kb) [file 12866_2015_537_MOESM3_ESM.pdf]
